# How Dry I Am!

**Published:** 1909-10-15

**Authors:** George Zederbaum

**Affiliations:** Charlotte, Mich.


### "HOW DRY I AM!"

BY GEORGE ZEDERBAUM, D.D.S., CHARLOTTE, MICH.

On the 5th day of April 1909, nineteen more prohibition counties in Michigan were added to eleven already in existence. Not being fully satisfied with this grand local option wave, a bill, known as the "Search and Seizure" bill was introduced by Mr. Dickinson, and passed during the last session of the Michigan legislature and became operative on the first day of September, 1909. Like the proverbial straw that broke the camel's back, this is certainly stretching the point to its breaking. I wish to quote only a part of sections No. 1 and No. 26 and show wherein the dental profession has been absolutely ignored, either willfully or negligently, and is placed on a par with merchants or laborers who have no concern with the treatment of diseases of the human body. Section 1 in part, says: "It shall be unlawful for any person directly or indirectly to manufacture, sell, keep for sale, *give away or furnish*, any vinous, malt, brewed, fermented, spirituous or intoxicating liquors or any beverages *any part of which is intoxicating*, provided however that the provision of this section shall not apply to druggist or registered pharmacist selling such liquors under and in compliance with the restriction and requirements imposed upon them by the general laws of this State and section No. 26 of this act as amended."

Section No. 26 says in part: "Every druggist keeping a drugstore in any county adopting prohibition under this act shall by himself or his clerk be permitted to sell such vinous, malt, brewed, fermented, spirituous and intoxicating liquors for *medicinal purposes*, but only on the written (not printed or typewritten) prescription of a *regular practicing physician*, provided that the *physician* making such prescription shall state therein the name of the person for whom such liquor is prescribed, and shall issue the same in good faith and upon *personal knowledge* that the physical condition of the person for whom such liquor is prescribed, requires the same for *medicinal purposes*."—The same section No. 26, further says: "Every such druggist so keeping a drugstore as aforesaid, shall be permitted to sell pure alcohol, or any vinous, fermented or spirituous liquors for art, chemical, scientific or mechanical purposes, (*not medicinal*) but only on the written application of the purchaser stating the purpose for which such liquors are purchased, which application shall be *subscribed and sworn* to by the person making such purchase."

Now this act is all very well to the general public, but how are dental practitioners going to be affected? Of course the law might be "stretched"—The following standard definitions being found for the word "physician": "A person skilled in the art of healing; one whose profession is to prescribe remedies for diseases." (Webster.) "One who is skilled in or practices art of healing. One who, being duly qualified, prescribes remedies for diseases." (The American Encyclopedic Dictionary.) "One whose vocation is the alleviation and cure of disease by therapeutic agencies. It includes the surgeon who conducts any surgical operation or treatment essential to life or comfort." (E. Darwin Hudson Jr.), and there are many more like definitions all agreeing in the main that a physician is not necessarily an M. D. This plan however, has been frustrated by the office of attorney general who has given the following decision in case of challenged prescription in Osceola, one of the local option counties.

Lansing, Mich.
Sept. 3rd. 1909.

Mr. Fred Trumbull, Prosecuting Attorney, Evart, Mich.

Dear Sir:—"I am in receipt of your two letters of the 31st. ultimo, and the 1st. instant respectively, in which you submit the questions—can liquor be lawfully sold by druggists on prescription of dentists and veterinary surgeons in option counties. In reply thereto will say that Sections 25 and 26 of Act 107 of the Public Acts 1909 prescribe the method by which liquors can be sold by druggists for medicinal purposes, and these sections limit the sale to persons presenting a prescription from a regular practicing physician or the written and signed application of the superintendent of any hospital, medical or educational institution where liquors are used only for medical, scientific purposes, and also permit the sale for art, chemical, scientific or mechanical purposes. The only way that intoxicating liquors can be purchased for medicinal purposes in local option counties, is upon the prescription of a regular practicing physician. Dentists and Veterinary surgeons are not regular practicing physicians unless they are legally qualified as such. It is our opinion that intoxicating liquors cannot be sold in local option counties upon the prescription of veterinary surgeons and dentists. Very respectfully,

(Signed) Henry E. Chase,

Deputy Attorney General.

The Deputy Attorney General was kind enough to transmit a copy of the above in answer to my inquiry of September 7th in which I addressed to the office of Attorney General as follows:—.

Dear Sir:—I write for the Dental profession of Michigan, for information relative section No. 26 Senate enrolled act No. 48, which allows only a regular practicing physician to write prescriptions for spirituous liquors, etc. Webster gives the following definition for the word "Physician" "A person skilled in the art of healing; one whose profession is to prescribe remedies for diseases." The dental profession is skilled in the art of healing of dental organs, oral cavities in general, often the tongue, the tonsils, the palates, the gums, the arches are legally treated by the dental surgeon, therefore the Dental profession comes under the category of the word physician as defined by Webster and as prescribed by the section mentioned. The Dental profession legitimately administers local and general anaesthetics and is very often called upon to prescribe brandy or other similar quickly acting stimulants. Are we to hunt up a physician, and have him write a prescription at a hazard of a total collapse or even death of the patient, while so doing, or can we legally write a prescription for it as we would for any other drug under the canopy of heaven?

I would sincerely thank you for your opinion on this question and this opinion, if you do not object, will be published in the official organ of the Michigan State Dental Society.

Yours very respectfully,

(Signed) George Zederbaum, D. D. S.

Editor of Transactions.

The reply received the following day was:

"Mr. George Zederbaum,

Charlotte, Mich.

Dear Sir:—Yours of the 7th. received. I enclose you a copy of an opinion given to Fred Trumbull, Prosecuting Attorney, Evart, Mich., under date of September 3rd 1909.

Yours respectfully,

(Signed) Henry E. Chase,

Deputy Atty. Genl.

Several good legal authorities say the act would not stand law in this respect, there being a material difference between a "*Dentist,*" a man who simply passes the State Board, and a man who first obtained the degree of *Doctor of Dental Surgery* from a recognized institution. But in the meanwhile what are we to do in these dry places? The law is not only irrational but as I stated before, may place some legal practitioner of our profession in a serious predicament. The law does not even take care of men practicing dentistry who have both the M. D. and the D. D. S. degrees for they can not according to the opinion given, be classed as *regular practicing physicians.* But why should there be such a reprehensible law, belittling our noble calling? The greater majority of dentists are not drunkards; neither do they sympathize with drunkards or the traffic in intoxicating liquors.

A dental practitioner abusing himself by drink or assisting in promulgating this traffic would soon be unable to perform the delicate operations demanded in the practice of dentistry. In spite of the fact that dentistry has been repeatedly legally classed as a profession, and in spite of the fact that in Michigan we can legally write prescriptions for Morphine (a dangerous drug which very few of us ever use), and cocaine, we are now placed in the category of merchants and saloon keepers. I do not see how we can legally obtain the necessary liquors and alcohol necessary in the practice of our profession. If we did obtain alcohol for use in the alcohol lamp, we should be trespassing upon this law, should we attempt to use it for any other purpose about the mouth. Ludicrous is it not? Again, many practitioners prescribe "Liquor Antisepticus," an official preparation and a good mouth wash indeed, yet, under this act we can not legally prescribe it, since in 1000 parts of this "Liquor" 250 parts of alcohol are used. Further (this sounds like the third degree), imagine yourself a legally practicing D. D. S. running to find a physician, who can not give you a prescription for your patient without seeing him personally and incidentally subjecting him or you to an additional fee for a prescription! Meanwhile what of the patient? Why does our State University have in the dental curriculum such studies as Physiology, Histology, Chemistry, General and Special Materia Medica and Therapeutics, Special Pathology, Oral Surgery, Anaesthesia, etc. all required for the degree of Doctor of Dental Surgery? If the graduates of that school are not qualified to prescribe, why should the State Board examine candidates for dental licenses relative to these subjects? Why license a D. D. S. if we are to be driven back 75 years to the days when the blacksmith and the barber wielded the turn-key? I wonder how long Senator Dickinson or any other learned Solon could stand on ceremony if he were suffering from dental troubles such as we see every day, demanding the use of liquor or alcohol? A committee connected with our State Society should look after this and all legislation and guard against any such infringement on our rights. The old legal definition of a "dentist", is one whose profession is to clean and extract teeth, repair them when they are diseased, and replace them with artificial ones when necessary." It does not hold good now, it is insufficient and should be revised. Dentistry made greater strides within the last twenty years than any other profession; in fact it made these strides so rapidly that most of our agricultural law makers have not kept up with them. I wonder what effect a few dental journals and text books would have if we could distribute them to each member of the Senate? The law as it now stands, ignores and degrades the profession of Dentistry. We must turn the wheels of the next legislature in our direction, but until then—"How Dry I Am—Nobody Knows How Dry I AM!"

